# Supplementary material for: Integration of AIEgens into covalent organic frameworks for pyroptosis and ferroptosis primed cancer immunotherapy
Source: Nat Commun. 2023 Sep 2;14:5355. doi: 10.1038/s41467-023-41121-z (PMC10475094; doi:10.1038/s41467-023-41121-z)
Supplement: Supplementary file 1 — Supplementary Information [file 41467_2023_41121_MOESM1_ESM.pdf]

Supplementary Information

Integration of AIEgens into Covalent Organic Frameworks for Pyroptosis and Ferroptosis Primed Cancer Immunotherapy

Liang Zhang[1,2,4,5]*, An Song*[1,5]*, Qi-Chao Yang*[1]*, Shu-Jin Li*[1]*, Shuo Wang*[1]*, Shu-Cheng Wan*[1]*, Jianwei Sun*[2]*, Ryan T. K. Kwok*[2]*, Jacky W. Y. Lam*[2,*]*, Hexiang Deng*[4,*]*, Ben Zhong Tang*[2,3,*]*, Zhi-Jun Sun*[1,*]

[1] State Key Laboratory of Oral & Maxillofacial Reconstruction and Regeneration, Key Laboratory of Oral Biomedicine Ministry of Education, Hubei Key Laboratory of Stomatology, School & Hospital of Stomatology, Wuhan University, Wuhan 430079, China.

[2] Department of Chemistry, and The Hong Kong Branch of Chinese National Engineering Research Center for Tissue Restoration and Reconstruction, The Hong Kong University of Science and Technology, Clear Water Bay, Kowloon, Hong Kong, 999077, China.

[3] Shenzhen Institute of Aggregate Science and Technology, School of Science and Engineering, The Chinese University of Hong Kong, Shenzhen, Guangdong 518172, China.

[4] Key Laboratory of Biomedical Polymers-Ministry of Education, College of Chemistry and Molecular Sciences, Wuhan University, Luojiashan, Wuhan 430072, China.

[5]These authors contributed equally: Liang Zhang, An Song.

*Email: chjacky@ust.hk; hdeng@whu.edu.cn; tangbenz@cuhk.edu.cn; sunzj@whu.edu.cn

The PDF file includes:

Supplementary Figures 1-23

Supplementary Tables 1-3

Supplementary Methods

Chemicals: The organic solvent was procured from Adamas-beta®, while 2,7-dichloro-dihydrofluorescein diacetate (DCFH-DA) was obtained from Jilin Chinese Academy of Sciences-Yanshen Technology Co., Ltd., and $N^1,N^{1'}$-(1,4-phenylene)bis(N^1-(4-aminophenyl)-benzene-1,4-diamine) (M-TPA) was bought from Shanghai Kylpharm Co., Ltd.. Puromycin, fetal bovine serum (FBS), penicillin/streptomycin (PS), and RPMI-1640 medium were sourced from Thermo Fisher (MA, USA). The CCK8 was acquired from Dojindo Laboratories (Japan), while the anti-mouse PD-1 mAb (CD279) was purchased from BioXcell (NH, USA).

Instruments: Fluorescence spectra were obtained using Cary Eclipse Fluorescence Spectrophotometer (Agilent Technologies). UV-vis absorption spectra were obtained using Cary 300 UV-vis Spectrophotometer (Agilent Technologies). Size distribution and zeta potential were detected by using Zetasizer Nano-ZS90 (Malvern). XPS was recorded by Thermal Fisher ESCALAB 250Xi. ICP measurements were performed on a THERMO Intrepid XSP Radial ICP-AES instrument.

Synthesis of COF@PEG sample: These COF samples were well dispersed into PBS solution using F-127 as PEG gens. Typically, 100 mg of COF samples and 200 mg F-127 were dispersed in 100 ml PBS and then sonicated via an 1800 W ultrasonic cell shredder (JY99-IIDN, from Ningbo Scientz Biotechnology Co., Ltd, www.scientz.com) for 24 h. The resulting mixture was centrifuged at 1000 × g for 30 min to discard un-isolated nanoparticles and then centrifuged at 30000 × g for 20 min to obtain the isolated COF@PEG samples.

FT-IR spectra for ligands and COFs: All IR spectra were recorded on a Thermo Nicolet iS10 IR spectroscopy made by Thermo Fisher, and samples were tableted and utilized KBr background. The wavelength range is 3800 to 500 cm^{-1}. The successful formation of imine linkage was evidenced by the emergence of a 1620 to 1622 cm^{-1} peak in the Fourier transform infrared (FT-IR) spectrum, representing the telescopic vibration of the imine bond ($\nu_{C=N}$).

X-ray Photoelectron Spectrometer (XPS) measurement of the molecular chromophores and their corresponding COFs: X-ray Photoelectron Spectrometer (XPS) was carried out on a Thermo Fisher ESCALAB 250Xi spectrometer, with the ion source of Al Kα, and samples were prepared in tablet for the test. The XPS spectra reflected the change in the chemical environment of nitrogen before and after the conversion to COFs. New N 1*s* peaks were observed at 398.9 eV, indicating the successful formation of imine bonds.

***UV-Vis diffuse reflectance spectra for ligands and COFs*:** UV-vis DRS of COFs were performed on a PerkinElmer Lambda 750 S spectroscopy using $BaSO_4$ as background. Band gap calculations were based on the Kubleka-Munk theory. The optical absorption coefficient (α) was calculated from reflectance data according to the Kubelka-Munk equation that $F(R) = \alpha = (1-R)^2/2R$, in which R is the percentage of reflected light. The incident photon energy (hν) and the optical band gap energy (Eg) are related to the transformed Kubelka-Munk function, $[F(R)h\nu]^2 = A(h\nu-Eg)$, where Eg is the band gap energy, A is the constant depending on transition probability, and intercept of $[F(R)h\nu]^2$ vs hν is Eg. In comparison to the corresponding molecular building blocks, these COFs exhibited smaller band gaps.

N_2 adsorptions analysis of these COFs: N_2 adsorptions isotherms were measured on a Quantachrome Autosorb-IQ2 automatic volumetric instrument in a liquid nitrogen bath (77 K). Ultra-high purity grade N_2 was used for the adsorption experiments. The BET surface area analysis was performed by plotting $x/v(1 - x)$ vs x, where $x = P/P_0$ (P_0 = 1 bar) and v is the volume of nitrogen adsorbed per gram of COF at standard temperature and pressure (STP), where satisfactory correlation coefficients and positive C constant were observed. The slope ($[c - 1]/v_m c$) and y-intercept ($1/v_m c$) of the linear region between the dashed lines give the monolayer capacity, v_m, that is then used to calculate the surface area from $A = v_m \sigma_0 N_{AV}$, where σ_0 is the cross-section area of the adsorbate at liquid density (16.2 Å^2 for nitrogen) and N_{AV} is Avogadro's number. The surface areas of these COFs range from 780 to 870 m^2 g^{-1}.

Determine the crystal structure of these COFs: Powder X-ray diffraction (PXRD) characterization was performed on a Smart lab diffractometer (Rigaku) with filtered Cu Kα radiation. Molecular models of COF-818 and COF-919 were generated with the Materials Studio (ver. 8.0) suite of programs. Pawley refinement was performed using Reflex, a software package for crystal determination from the PXRD pattern. Unit cell dimension was set to the theoretical parameters. The Pawley refinement was performed to optimize the lattice parameters iteratively until the Rwp and Rp value converges and the overlay of the observed with refined profiles shows good agreement. The lattice models were then fully optimized using MS Forcite molecular dynamics module method for each COF model. We performed Pawley refinement using the Reflex Plus module of the Material Studio, which showcases good agreement with the experimental PXRD patterns, with (Rwp, Rp) values of (1.11%, 0.85%) for COF-818, (Rwp, Rp) values of (0.58%, 0.47%) for COF-919 with Pawley refined cell parameters of $\boldsymbol{a}$ = 28.03 Å, $\boldsymbol{b}$ = 22.59 Å, $\boldsymbol{c}$ = 3.72 Å and $\alpha = \beta = \gamma = 90°$, for COF-818, $\boldsymbol{a}$ = 27.39 Å, $\boldsymbol{b}$ =

21.73 Å, ***c*** = 3.91 Å, $\alpha = \beta = \gamma = 90°$ for COF-919.

DFT calculations: The specific process conformational search is to use ORCA5.0.3 package[1], and the first excited state geometry optimization was conducted under the wB97X-D3/def2SVP level[2-3].

Cellular iron assay: 4T1 cells were incubated with COF samples (100 μg mL^{-1}) for 24 h. The resulting samples were subjected to different treatments and irradiated with 660 nm (0.5 W cm^{-2}, 5 min) and/or 808 nm (1.5 W cm^{-2}, 5 min) lasers. After the 6 h treatment, cells were washed and used for measurement of cellular iron assay. The intracellular ferrous iron (Fe^{2+}) level was determined using an iron assay kit, as per the manufacturer's instructions. Briefly, cells were seeded onto a plate with 5×10^6 cells per plate, collected, and washed in ice-cold phosphate buffer solution. Next, 100-200 μl of lysate was added, and the mixture was centrifuged at 4 °C. The supernatant was then treated with an iron reducer and incubated at 60℃ for 60 min. Subsequently, 30 μl of the iron probe was added, and the solution was mixed thoroughly using a horizontal shaker and incubated at 25 °C for 30 min. Finally, the 550 nm absorbance peak was measured using a microplate reader.

Mitochondrial morphology assays: 4T1 cells were incubated with COF samples (100 μg mL^{-1}) for 24 h. The resulting samples were subjected to different treatments and irradiated with 660 nm (0.5 W cm^{-2}, 5 min) and/or 808 nm (1.5 W cm^{-2}, 5 min) lasers. After the 6 h treatment, cells were washed with PBS three times and fixed in 4% paraformaldehyde solution for 1 hour, and the mitochondrial morphology was analyzed by TEM. The cells were fixed in 0.1 M sodium cacodylate buffer solution (pH 7.4) with

2.5% glutaraldehyde, followed by post-fixation in 2% aqueous osmium tetroxide and dehydration in an ethanol/propylene oxide mixture. The cells were then embedded in Epon solution for 24 h at 60 °C (Merck, Darmstadt, Germany). Prior to TEM analysis, the cells were cut into ultra-thin sections, stained with 0.5% uranyl acetate for 30 min, and then further stained with 3% lead citrate for 7 min.

Supplementary References

1 Neese, F., et al. Electronic structure and spectroscopy of novel copper chromophores in biology. Konstanz: University of Konstanz; 1997.

2 Chai, J.-D., et al. Systematic optimization of long-range corrected hybrid density functionals. *J. Chem. Phys.* **128**, 084106-084130 (2008).

3 Grimme, S., et al. A consistent and accurate ab initio parametrization of density functional dispersion correction (DFT-D) for the 94 elements H-Pu. *J. Chem. Phys.* **132**, 154104-154123 (2010).

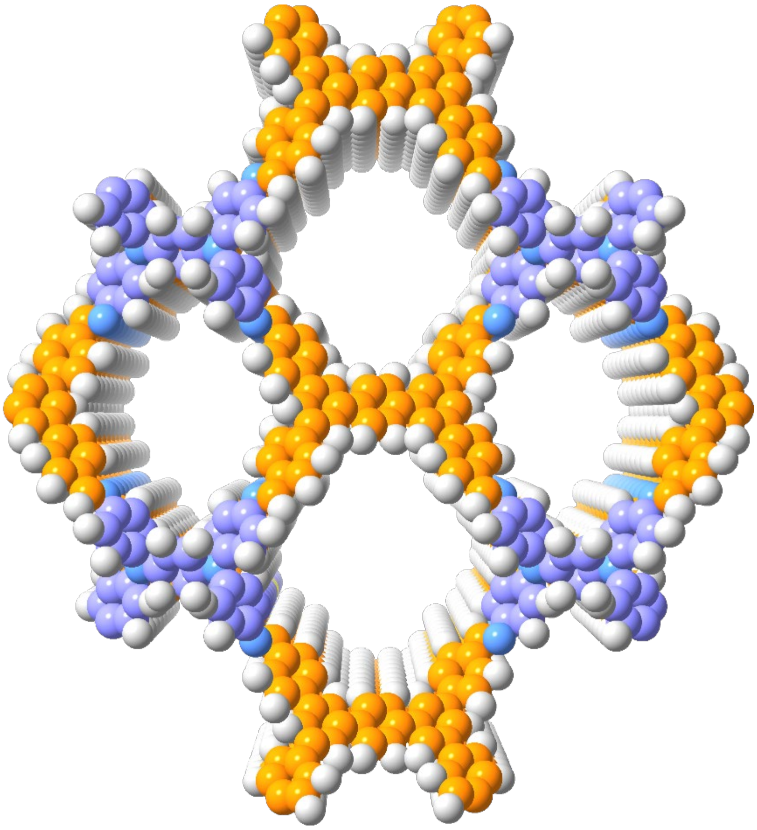

Supplementary Fig. 1. Space-filling models of COF-818 from the top view.

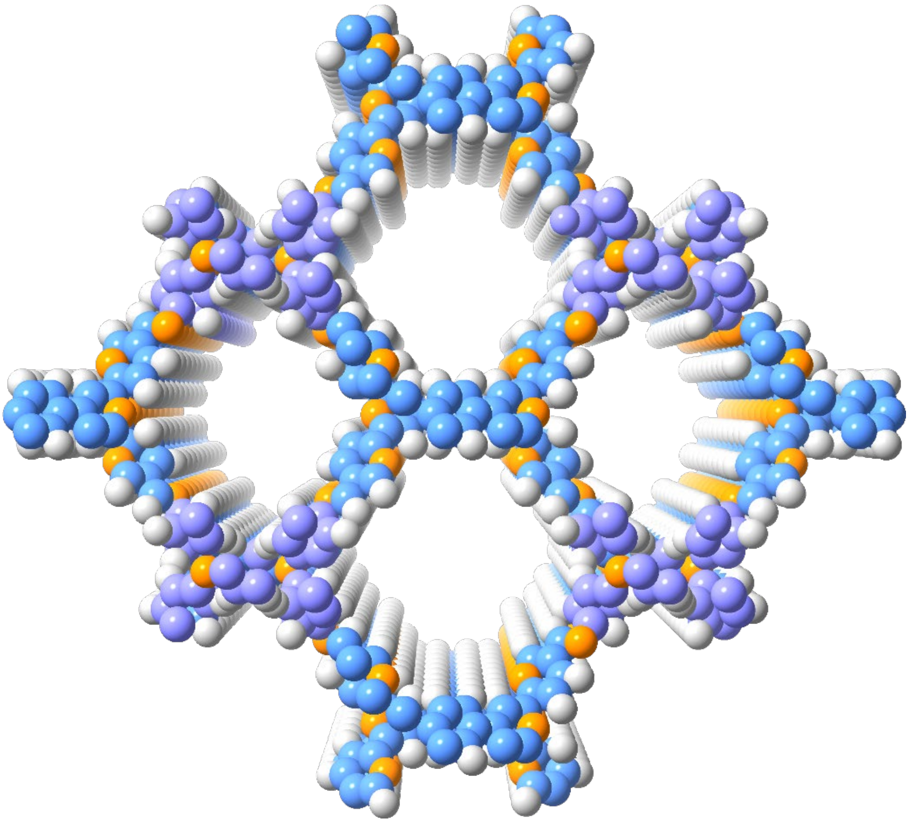

Supplementary Fig. 2. Space-filling models of COF-919 from the top view.

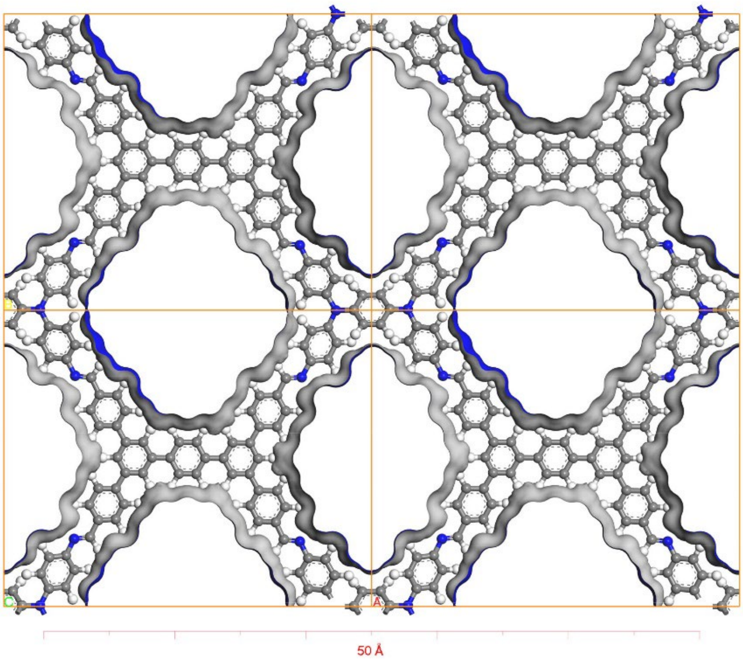

Supplementary Fig. 3. Connolly surface of COF-818, carbon, hydrogen and nitrogen atoms are represented as grey, white, and blue spheres, respectively.

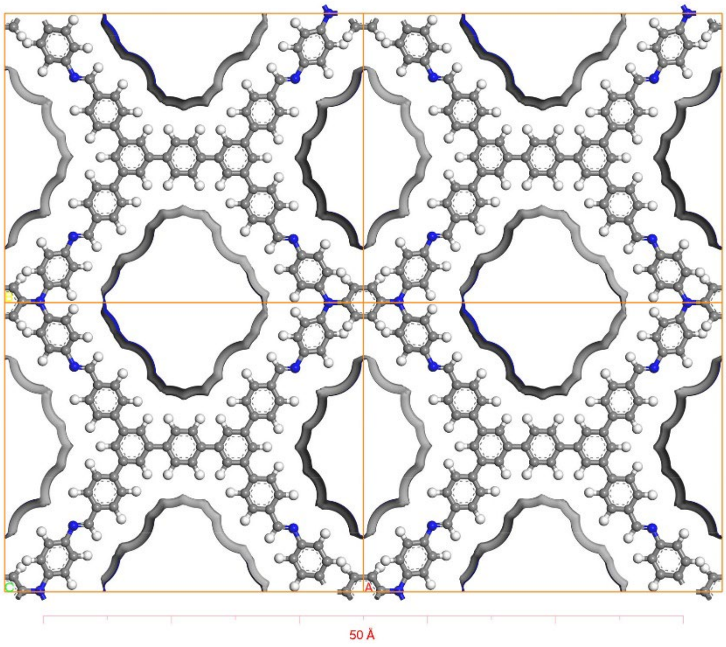

Supplementary Fig. 4. Solvent surface of COF-818, carbon, hydrogen and nitrogen atoms are represented as grey, white, and blue spheres, respectively.

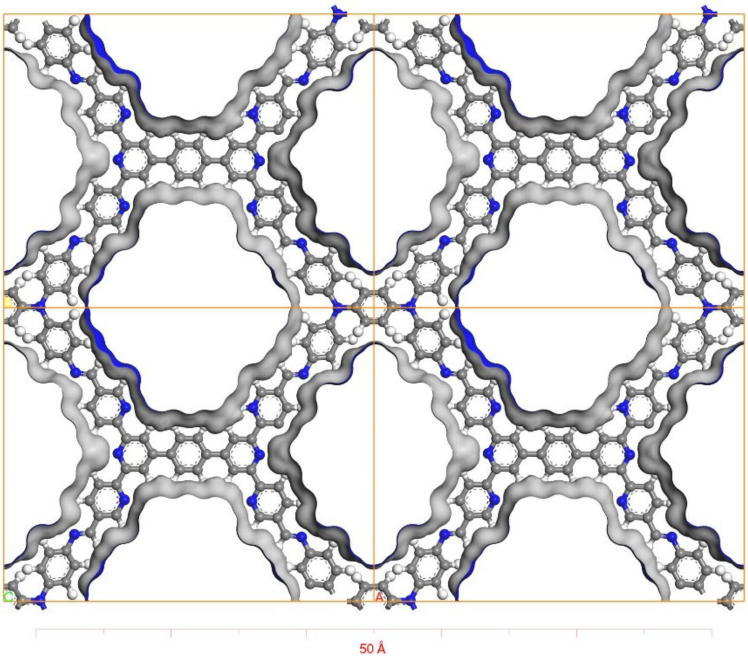

Supplementary Fig. 5. Connolly surface of COF-919, carbon, hydrogen and nitrogen atoms are represented as grey, white, and blue spheres, respectively.

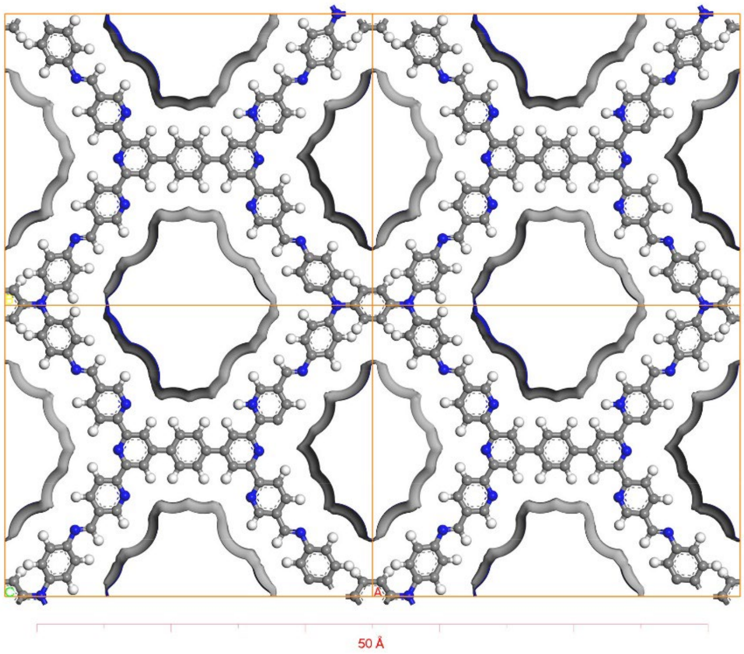

Supplementary Fig. 6. Solvent surface of COF-919, carbon, hydrogen and nitrogen atoms are represented as grey, white, and blue spheres, respectively.

Supplementary Table 1. Crystallographic Information of Modeled COFs.

	**COF-818**	**COF-919**
**Space group**	*Cmmm*	*Cmmm*
a **(Å)**	28.03	27.39
b **(Å)**	22.59	21.73
c **(Å)**	3.72	3.91
R_p **(%)**	1.11	0.58
R_{wp}**(%)**	0.85	0.47
α (°)	90	90
β(°)	90	90
γ(°)	90	90

Supplementary Table 2. Atomic parameters of COF-818

Atom	*x* (Å)	*y* (Å)	*z* (Å)	Atom	*x* (Å)	*y* (Å)	*z* (Å)
**Atomic parameters of COF-818**							
C1	0.32594	0.34581	-0.04425	C32	0.47669	0.55476	0.02905
C2	0.30363	0.29411	-0.06502	C33	0.67003	0.34052	-0.07718
C3	0.7659	0.65991	-0.00925	C34	0.69191	0.28797	-0.08823
C4	0.30425	0.39753	-0.0125	C35	0.23611	0.66352	0.10739
C5	0.25664	0.29161	-0.0541	C36	0.69276	0.39166	-0.0508
N6	0.80966	0.77384	-0.02624	C37	0.73909	0.28431	-0.07152
C7	0.02409	0.95247	0.23996	N38	0.19385	0.77867	0.10378
C8	0.90446	0.89009	-0.10583	C39	0.97703	0.95271	0.22266
C9	0.88125	0.83352	-0.10004	C40	0.10133	0.89555	-0.03713
C10	0.19134	0.12332	-0.12048	C41	0.12494	0.84087	-0.01118
C11	0.88098	0.94214	-0.03975	C42	0.80467	0.11398	-0.12374
C12	0.83421	0.82837	-0.02832	C43	0.12247	0.94566	0.05546
C13	0.76643	0.76982	0.01273	C44	0.17061	0.83436	0.1044
C14	0.66797	0.44742	-0.03214	C45	0.23732	0.77212	0.15368
C15	0.62081	0.44737	-0.02267	C46	0.33038	0.45176	0.01471
C16	0.52285	0.44784	0.0058	C47	0.37757	0.45054	0.01009
C17	0.67314	0.65925	0.02844	C48	0.47577	0.4483	0.01524
C18	0.69572	0.71146	0.03246	C49	0.32932	0.66027	0.13999
C19	0.23236	0.34311	-0.0236	C50	0.30739	0.71228	0.16082
C20	0.69499	0.60708	0.00595	C51	0.76435	0.33554	-0.04586
C21	0.74266	0.71294	0.01267	C52	0.30655	0.60959	0.10268
N22	0.19095	0.2281	-0.03032	C53	0.26026	0.71503	0.1435
C23	0.9745	0.04987	-0.23757	N54	0.80463	0.21921	-0.03568
C24	0.10073	0.11085	0.07441	C55	0.0242	0.05139	-0.22782
C25	0.12327	0.16579	0.05734	C56	0.89824	0.10517	0.05556
C26	0.81611	0.88067	0.02845	C57	0.87405	0.15862	0.04873
C27	0.12219	0.0601	-0.00522	C58	0.19317	0.8828	0.20501
C28	0.1685	0.17227	-0.03637	C59	0.87707	0.05438	-0.02823
C29	0.23412	0.23453	-0.06755	C60	0.82766	0.16346	-0.03906
C30	0.33153	0.55461	0.07254	C61	0.76114	0.22632	-0.07806
C31	0.37873	0.55465	0.06648	C62	0.66909	0.55174	-0.00251

Supplementary Table 2. Atomic parameters of COF-818

Atomic parameters of COF-818							
Atom	*x* (Å)	*y* (Å)	*z* (Å)	Atom	*x* (Å)	*y* (Å)	*z* (Å)
C63	0.62192	0.55281	0.00645	H98	0.398	0.59825	0.08733
C64	0.52378	0.5543	0.01943	H99	0.45821	0.59904	0.03875
C65	0.0492	0.00345	0.00701	H100	0.67192	0.24497	-0.11178
C66	0.5973	0.50035	-0.00317	H101	0.19767	0.66501	0.09413
C67	0.54738	0.50084	0.00758	H102	0.95677	0.91824	0.37899
C68	0.9518	7.3E-4	-0.01352	H103	0.06487	0.89627	-0.13581
C69	0.40223	0.5023	0.03543	H104	0.10729	0.79769	-0.08596
C70	0.45216	0.50177	0.02691	H105	0.76724	0.11673	-0.19358
C71	0.30876	0.50348	1.04574	H106	0.25943	0.81247	0.2088
C72	0.25922	0.61054	1.08778	H107	0.39612	0.40647	-0.01482
C73	0.25714	0.39856	0.99729	H108	0.45627	0.40475	0.01345
C74	0.69077	0.49929	0.97867	H109	0.32722	0.75525	0.19324
C75	0.74212	0.60678	0.98764	H110	0.80275	0.3329	-0.03293
C76	0.74261	0.38988	0.96394	H111	0.04452	0.08586	-0.38362
C77	0.82788	0.06009	0.87695	H112	0.93553	0.10512	0.13177
C78	0.16723	0.06615	0.89045	H113	0.89177	0.20205	0.11689
C79	0.17047	0.93753	1.18913	H114	0.22967	0.87904	0.30004
C80	0.83668	0.92967	1.02444	H115	0.74023	0.18452	-0.12174
N81	0.90284	0.99922	-0.02694	H116	0.60329	0.59695	0.02178
N82	1.09818	1.00316	0.01892	H117	0.54328	0.59785	0.02122
H83	0.32316	0.25081	-0.09234	H118	0.23986	0.56713	1.05926
H84	0.80434	0.65991	-0.02465	H119	0.23852	0.4426	1.02145
H85	0.04483	0.91944	0.40362	H120	0.76268	0.43287	0.98312
H86	0.94221	0.89194	-0.16493	H121	0.18546	0.02362	0.81076
H87	0.90076	0.79089	-0.15431	H122	0.19072	0.97746	1.28585
H88	0.22851	0.12633	-0.19823	H123	0.81199	0.96706	1.08668
H89	0.74527	0.8116	0.049	H124	0.36441	0.34639	-0.05355
H90	0.60156	0.40365	-0.03099	H125	0.36776	0.65912	0.15416
H91	0.54134	0.40356	-0.00346	H126	0.27027	0.50362	1.0492
H92	0.67629	0.75494	0.05225	H127	0.76107	0.5629	0.97014
H93	0.1939	0.34155	-0.01533	H128	0.82335	0.03973	0.59727
H94	0.95294	0.08239	-0.39828	H129	0.81138	0.02858	1.07836
H95	0.06367	0.10901	0.15689	H130	0.6347	0.65881	0.04431
H96	0.10432	0.20844	0.12259	H131	0.63159	0.34182	-0.09036
H97	0.25462	0.19242	-0.11286	H132	0.72925	0.49936	0.97191

Supplementary Table 3. Atomic parameters of COF-919

**Atomic parameters of COF-919**							
Atom	*x* (Å)	*y* (Å)	*z* (Å)	Atom	*x* (Å)	*y* (Å)	*z* (Å)
N1	0.32594	0.34581	-0.04425	C37	0.73909	0.28431	-0.07152
C2	0.30363	0.29411	-0.06502	N38	0.19385	0.77867	0.10378
C3	0.7659	0.65991	-0.00925	C39	0.97703	0.95271	0.22266
C4	0.30425	0.39753	-0.0125	C40	0.10133	0.89555	-0.03713
C5	0.25664	0.29161	-0.0541	C41	0.12494	0.84087	-0.01118
N6	0.80966	0.77384	-0.02624	C42	0.80467	0.11398	-0.12374
C7	0.02409	0.95247	0.23996	C43	0.12247	0.94566	0.05546
C8	0.90446	0.89009	-0.10583	C44	0.17061	0.83436	0.1044
C9	0.88125	0.83352	-0.10004	C45	0.23732	0.77212	0.15368
C10	0.19134	0.12332	-0.12048	C46	0.33038	0.45176	0.01471
C11	0.88098	0.94214	-0.03975	C47	0.37757	0.45054	0.01009
C12	0.83421	0.82837	-0.02832	C48	0.47577	0.4483	0.01524
C13	0.76643	0.76982	0.01273	N49	0.32932	0.66027	0.13999
C14	0.66797	0.44742	-0.03214	C50	0.30739	0.71228	0.16082
C15	0.62081	0.44737	-0.02267	C51	0.76435	0.33554	-0.04586
C16	0.52285	0.44784	0.0058	C52	0.30655	0.60959	0.10268
N17	0.67314	0.65925	0.02844	C53	0.26026	0.71503	0.1435
C18	0.69572	0.71146	0.03246	N54	0.80463	0.21921	-0.03568
C19	0.23236	0.34311	-0.0236	C55	0.0242	0.05139	-0.22782
C20	0.69499	0.60708	0.00595	C56	0.89824	0.10517	0.05556
C21	0.74266	0.71294	0.01267	C57	0.87405	0.15862	0.04873
N22	0.19095	0.2281	-0.03032	C58	0.19317	0.8828	0.20501
C23	0.9745	0.04987	-0.23757	C59	0.87707	0.05438	-0.02823
C24	0.10073	0.11085	0.07441	C60	0.82766	0.16346	-0.03906
C25	0.12327	0.16579	0.05734	C61	0.76114	0.22632	-0.07806
C26	0.81611	0.88067	0.02845	C62	0.66909	0.55174	-0.00251
C27	0.12219	0.0601	-0.00522	C63	0.62192	0.55281	0.00645
C28	0.1685	0.17227	-0.03637	C64	0.52378	0.5543	0.01943
C29	0.23412	0.23453	-0.06755	C65	0.0492	0.00345	0.00701
C30	0.33153	0.55461	0.07254	C66	0.5973	0.50035	-0.00317
C31	0.37873	0.55465	0.06648	C67	0.54738	0.50084	0.00758
C32	0.47669	0.55476	0.02905	C68	0.9518	7.3E-4	-0.01352
N33	0.67003	0.34052	-0.07718	C69	0.40223	0.5023	0.03543
C34	0.69191	0.28797	-0.08823	C70	0.45216	0.50177	0.02691
C35	0.23611	0.66352	0.10739	N71	0.30876	0.50348	1.04574
C36	0.69276	0.39166	-0.0508	C72	0.25922	0.61054	1.08778

Supplementary Table 3. Atomic parameters of COF-919

Atomic parameters of COF-919							
Atom	*x* (Å)	*y* (Å)	*z* (Å)	Atom	*x* (Å)	*y* (Å)	*z* (Å)
C73	0.25714	0.39856	0.99729	H100	0.398	0.59825	0.08733
N74	0.69077	0.49929	0.97867	H101	0.45821	0.59904	0.03875
C75	0.74212	0.60678	0.98764	H102	0.67192	0.24497	-0.11178
C76	0.74261	0.38988	0.96394	H103	0.19767	0.66501	0.09413
C77	0.82788	0.06009	0.87695	H104	0.95677	0.91824	0.37899
C78	0.16723	0.06615	0.89045	H105	0.06487	0.89627	-0.13581
C79	0.17047	0.93753	1.18913	H106	0.10729	0.79769	-0.08596
C80	0.83668	0.92967	1.02444	H107	0.76724	0.11673	-0.19358
N81	0.90284	0.99922	-0.02694	H108	0.25943	0.81247	0.2088
N82	1.09818	1.00316	0.01892	H109	0.39612	0.40647	-0.01482
H83	0.32316	0.25081	-0.09234	H110	0.45627	0.40475	0.01345
H84	0.80434	0.65991	-0.02465	H111	0.32722	0.75525	0.19324
H85	0.04483	0.91944	0.40362	H112	0.80275	0.3329	-0.03293
H86	0.94221	0.89194	-0.16493	H113	0.04452	0.08586	-0.38362
H87	0.90076	0.79089	-0.15431	H114	0.93553	0.10512	0.13177
H88	0.22851	0.12633	-0.19823	H115	0.89177	0.20205	0.11689
H89	0.74527	0.8116	0.049	H116	0.22967	0.87904	0.30004
H90	0.60156	0.40365	-0.03099	H117	0.74023	0.18452	-0.12174
H91	0.54134	0.40356	-0.00346	H118	0.60329	0.59695	0.02178
H92	0.63571	0.65882	0.0439	H119	0.54328	0.59785	0.02122
H93	0.67629	0.75494	0.05225	H120	0.23986	0.56713	1.05926
H94	0.1939	0.34155	-0.01533	H121	0.23852	0.4426	1.02145
H95	0.95294	0.08239	-0.39828	H122	0.76268	0.43287	0.98312
H96	0.06367	0.10901	0.15689	H123	0.18546	0.02362	0.81076
H97	0.10432	0.20844	0.12259	H124	0.19072	0.97746	1.28585
H98	0.77847	0.88459	0.08802	H125	0.81199	0.96706	1.08668
H99	0.25462	0.19242	-0.11286				

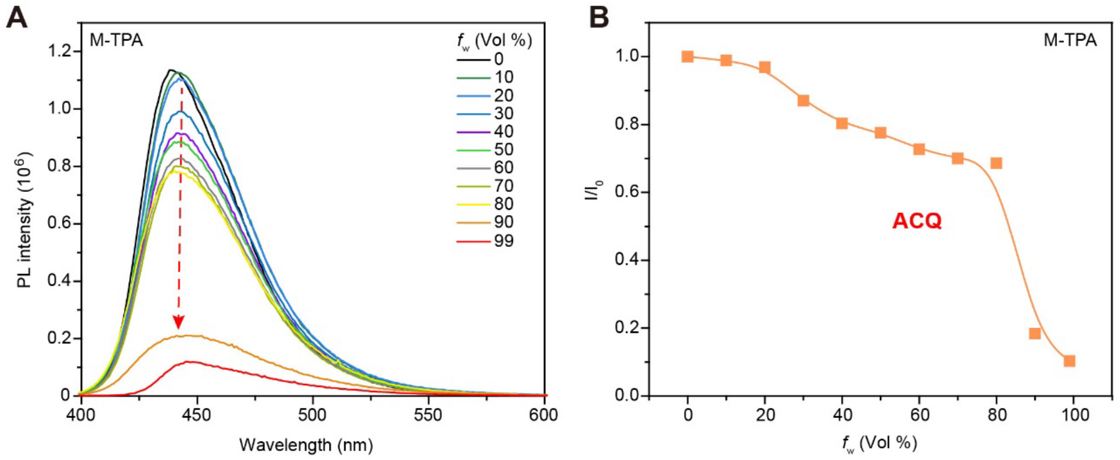

Supplementary Fig. 7. Aggregation-caused quenching (ACQ) effects of M-TPA, (A) photo luminescence spectrum with different water fractions. (B) ACQ curve of M-TPA. Source data are provided as a Source Data file.

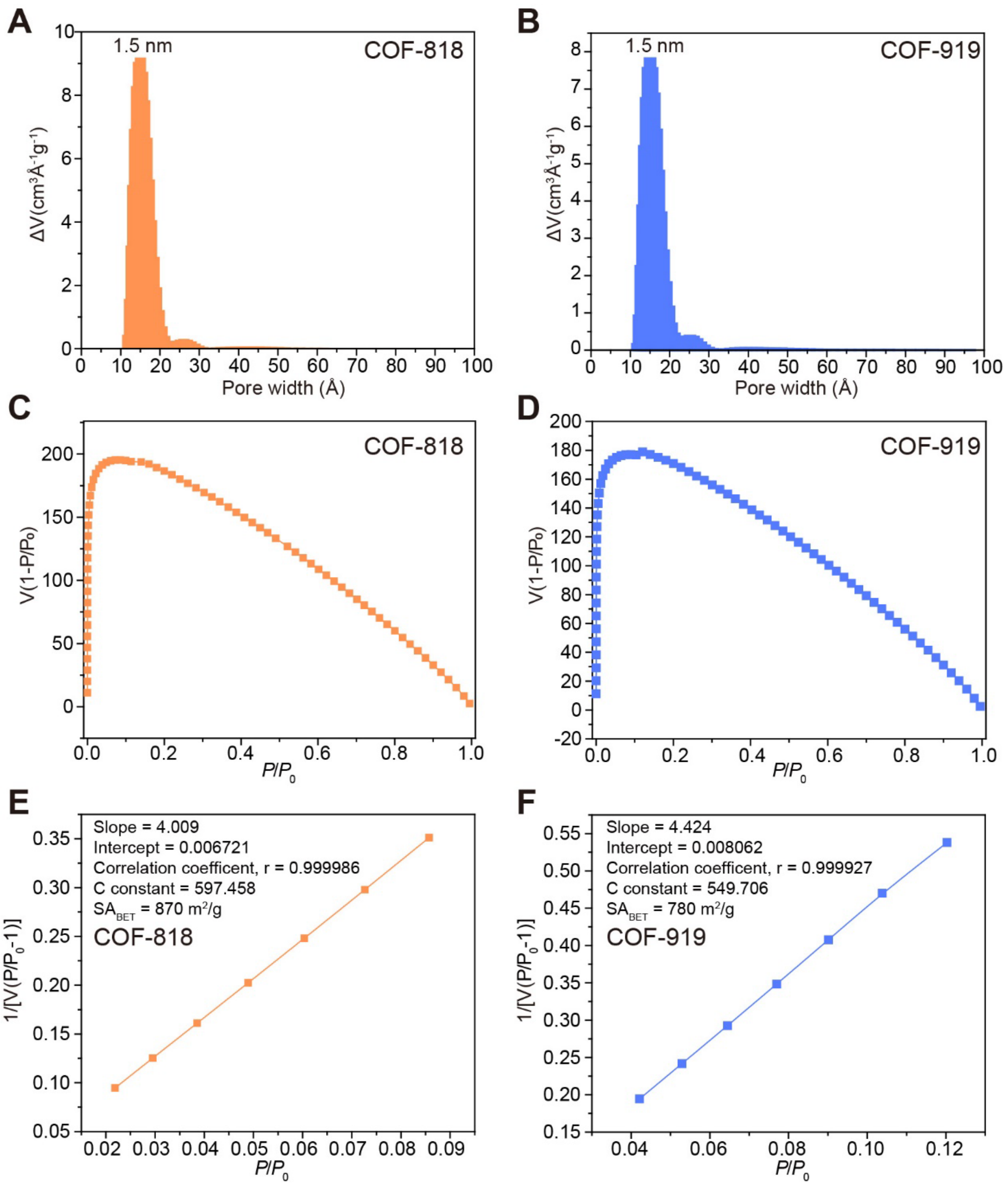

Supplementary Fig. 8. BET surface area was calculated for COF-818 and COF-919, based on nitrogen adsorption isotherm at 77 K. (A) and (B) pore size distribution of COF-818 and COF-919, (C) to (F) plot shows good fitting to the BET model. Source data are provided as a Source Data file.

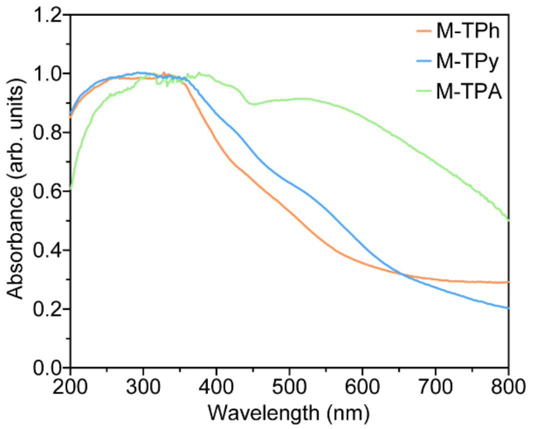

Supplementary Fig. 9. Solid UV-Vis absorbance spectra of monomers. Source data are provided as a Source Data file.

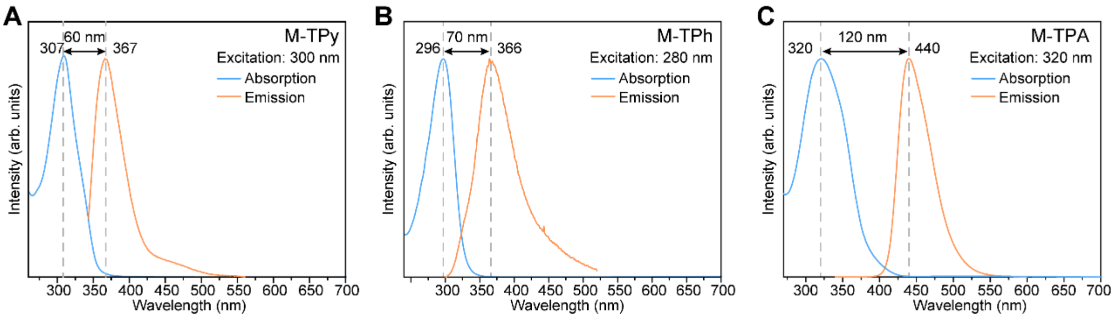

Supplementary Fig. 10. The UV-Vis spectra, fluorescence spectra, and Stokes shift of monomers, including M-TPy (A), M-TPh (B), and M-TPA (C). Source data are provided as a Source Data file.

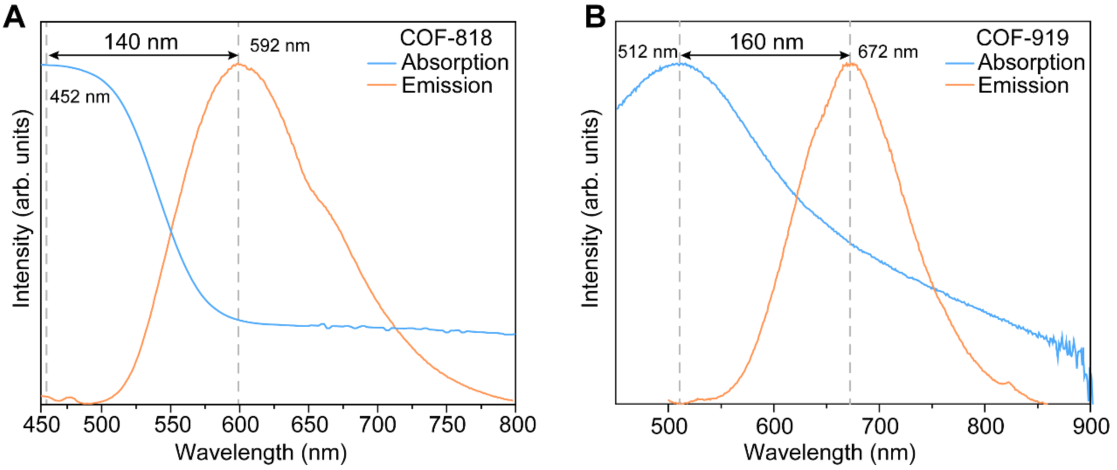

Supplementary Fig. 11. The UV-Vis spectra, fluorescence spectra, and Stokes shift of COF-818 (A) and COF-919 (B). Source data are provided as a Source Data file.

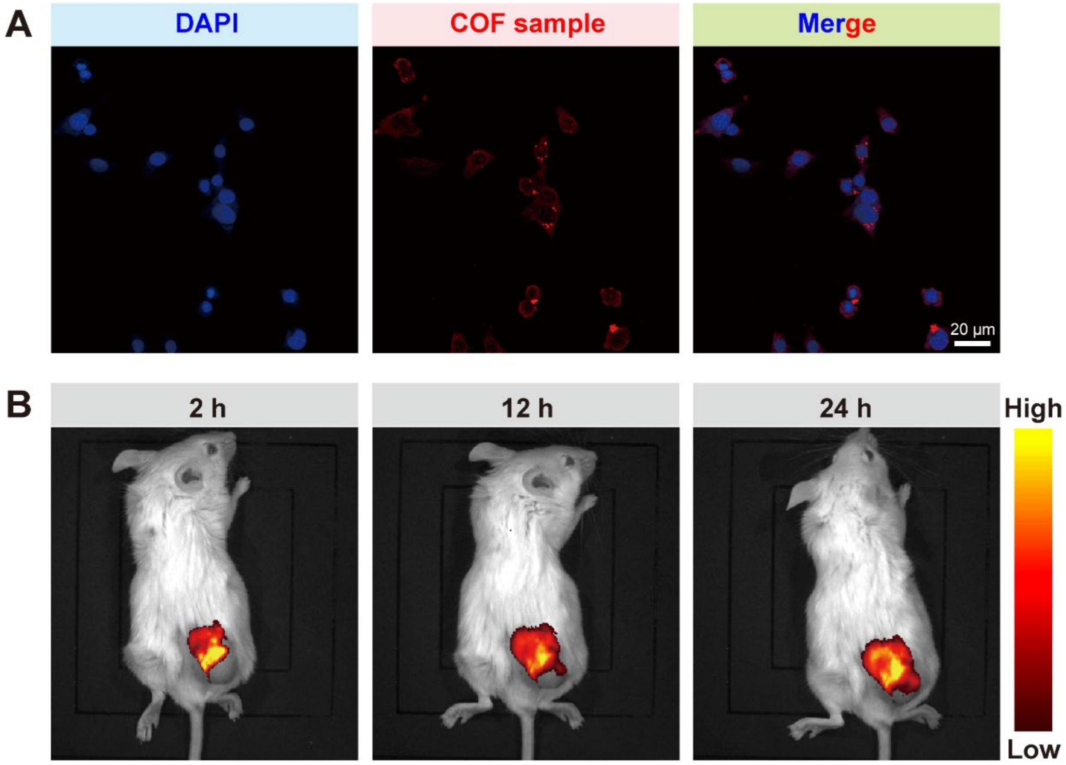

Supplementary Fig. 12. The in vitro bioimaging of COF-919 (A), the photographs of immunofluorescence staining were representative of those generated from three independent samples with similar results, and in vivo (B) bioimaging of COF-919.

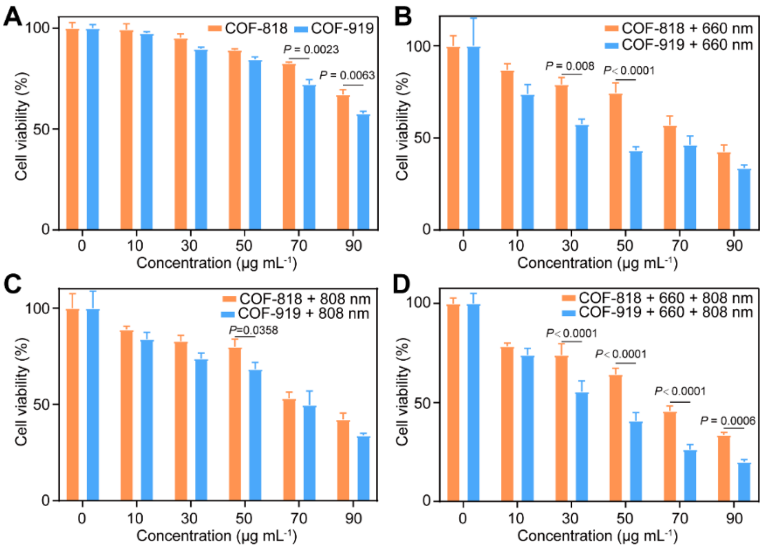

Supplementary Fig. 13. MTT assay of the viability of 4T1 cells treated with COF-818 and COF-919 at different conditions including: without laser irradiation (A); under 660 nm laser irradiation (B); under 808 nm laser irradiation (C); and under 660 nm plus 808 nm laser irradiation (D); all data are shown as the mean ± SD, $n = 3$ and n represents the number of independent samples. Statistical significance was calculated via two-tailed Student's *t*-test. Source data are provided as a Source Data file.

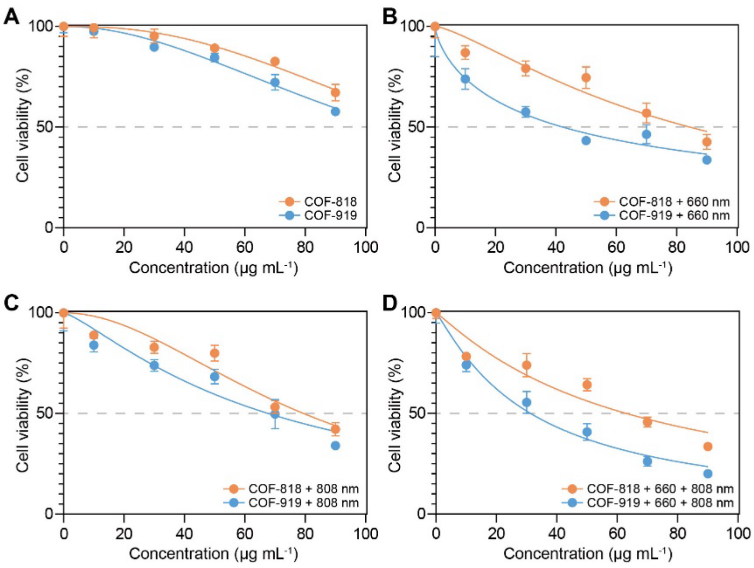

Supplementary Fig. 14. IC50 value of 4T1 cells treated with COF-818 and COF-919 at different conditions including: without laser irradiation (A); under 660 nm laser irradiation (B); under 808 nm laser irradiation (C); and under 660 nm plus 808 nm laser irradiation (D); all data are shown as the mean ± SD, *n* = 3 and n represents the number of independent samples. Source data are provided as a Source Data file.

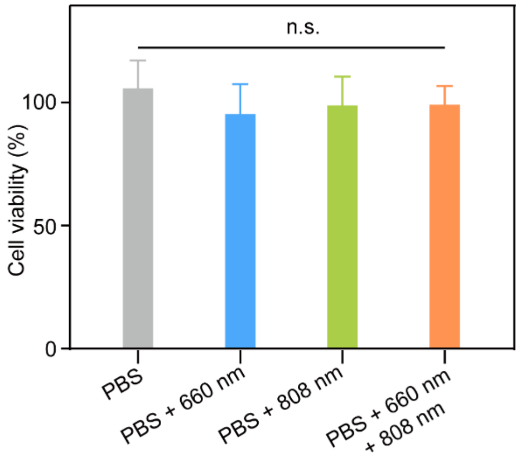

Supplementary Fig. 15. MTT assay of the viability of 4T1 cells treated with PBS, PBS + 660 nm, PBS + 808 nm, and PBS + 660 + 808 nm laser; all data are shown as the mean ± SEM, *n* = 3 and n represents the number of independent samples. Statistical significance was calculated via one-way ANOVA with Tukey's multiple comparisons test. Source data are provided as a Source Data file.

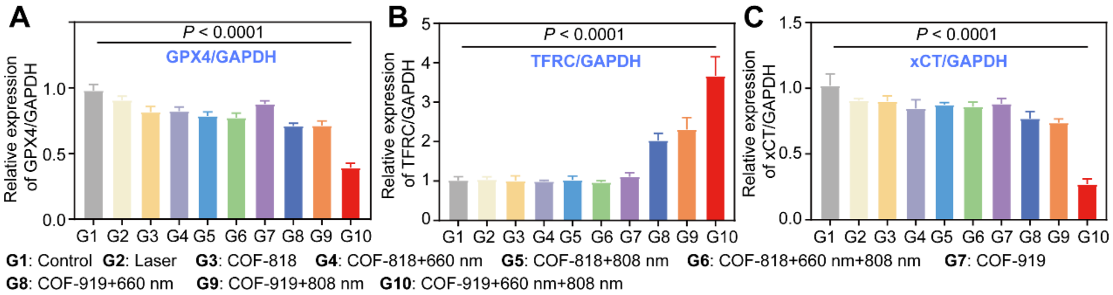

Supplementary Fig. 16. Western blot results of representative ferroptosis factors, including GPX4 (A), TFRC (B) and xCT (C) in 4T1 cells treated under different conditions; all data are shown as the mean ± SEM, $n = 3$ and n represents the number of independent samples. Statistical significance was calculated via one-way ANOVA with Tukey's multiple comparisons test. Source data are provided as a Source Data file.

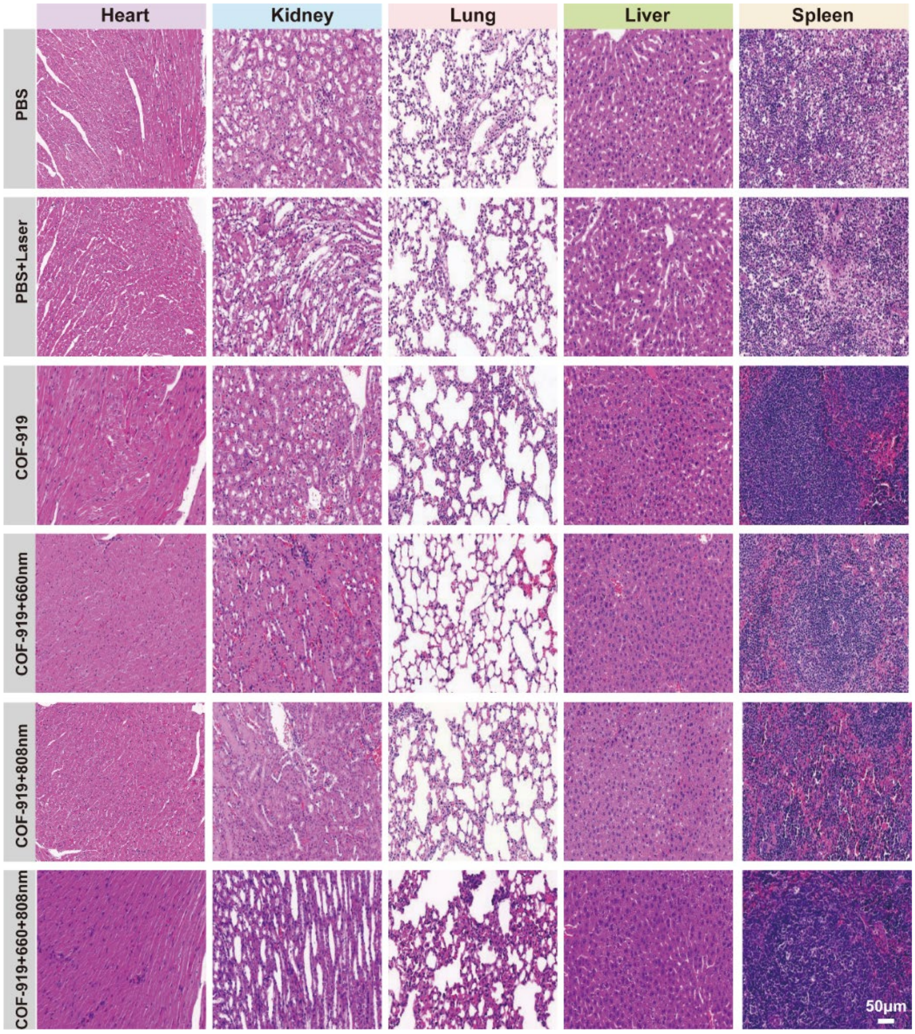

Supplementary Fig. 17. Representative H&E staining of major organs of differently treated mice, including hearts, livers, spleens, lungs, and kidneys, scale bar = 50 μm; the images of HE staining were representative of those generated from five mice each group with similar results.

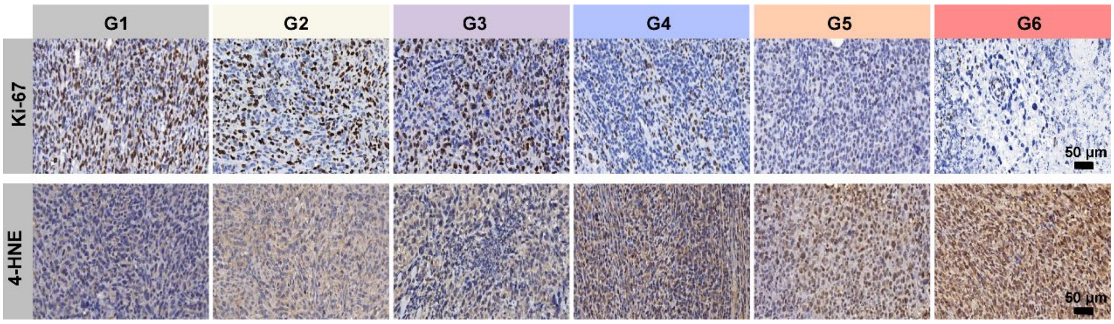

Supplementary Fig. 18. Representative 4-HNE and Ki-67 images of tumor tissues under different treatments, G1: Control; G2: 660+808 nm laser; G3: COF-919; G4: COF-919+660 nm; G5: COF-919+808 nm; G6: COF-919+660 nm +808 nm; scale bar = 50 μm; the images of immunostaining were representative of those generated from five mice each group with similar results.

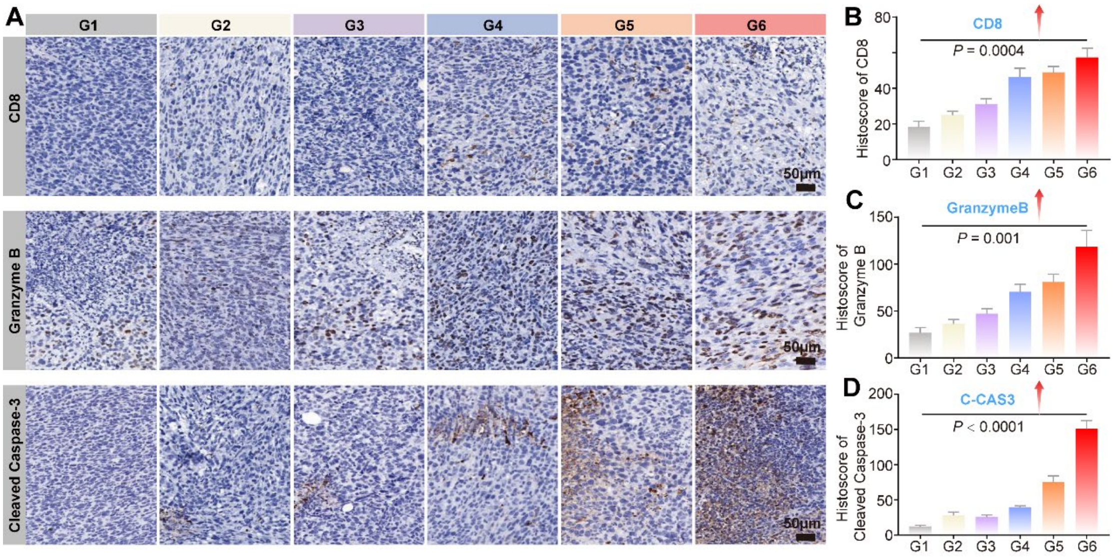

Supplementary Fig. 19. Representative immunohistochemical images of CD8, Granzyme B, and cleaved Caspase-3 under different treatments (A). Quantification and representative immunohistochemical images of CD8 (B), Granzyme B (C), and cleaved Caspase-3 (D) under different treatments. G1: Control; G2: 660+808 nm laser; G3: COF-919; G4: COF-919+660 nm; G5: COF-919+808 nm; G6: COF-919+660 nm +808 nm; scale bar = 50 μm; all data are shown as the mean ± SEM, *n* = 5 and n represents the number of independent samples. Statistical significance was calculated via one-way ANOVA with Tukey's multiple comparisons test. Source data are provided as a Source Data file.

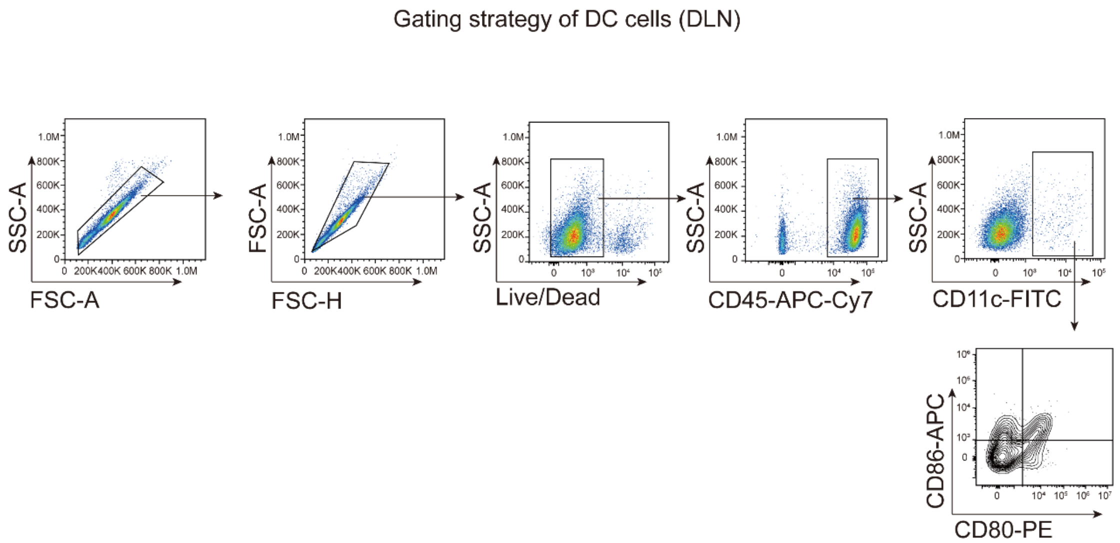

Supplementary Fig. 20. Gating strategy of activated DC cells in draining lymph nodes (DLN).

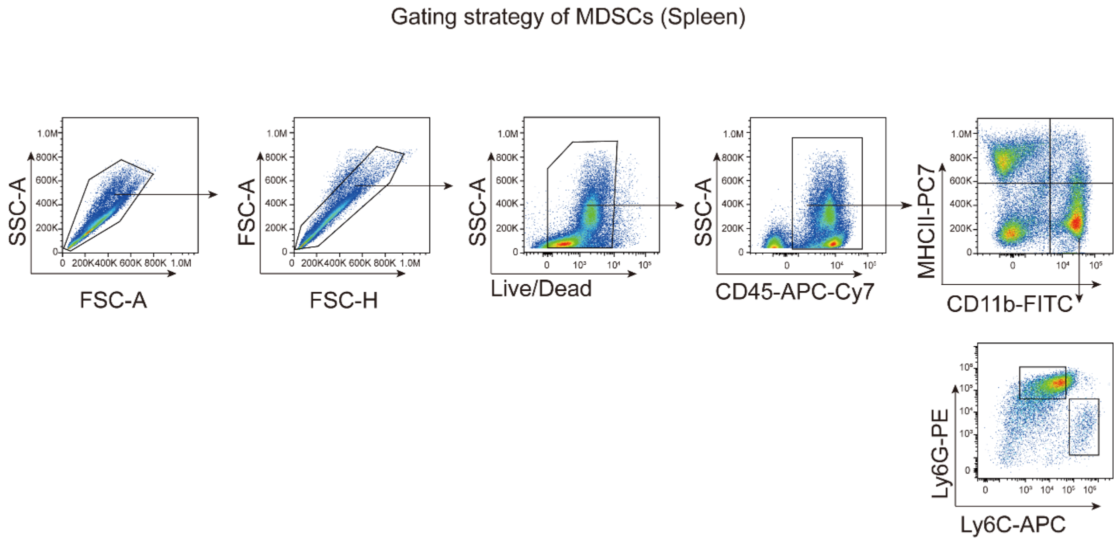

Supplementary Fig. 21. Gating strategy of myeloid-derived suppressor cells (MDSCs) in spleen.

Gating strategy of memory T cells

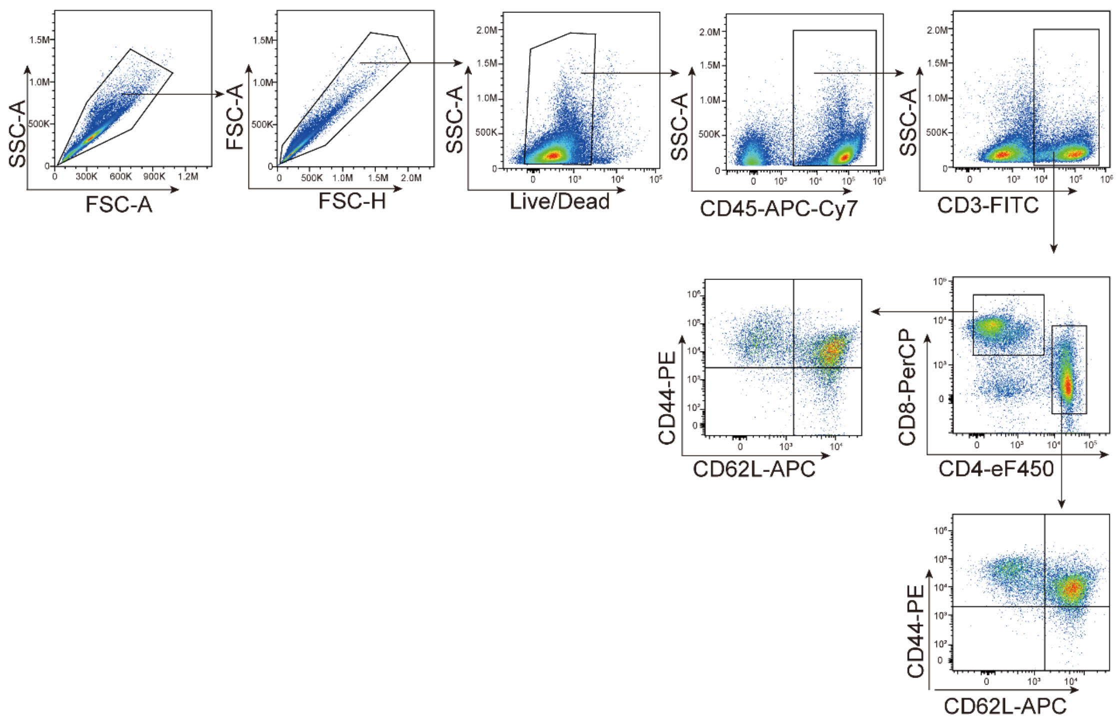

Supplementary Fig. 22. Gating strategy of memory T cells.

Gating strategy of Treg cells (DLN)

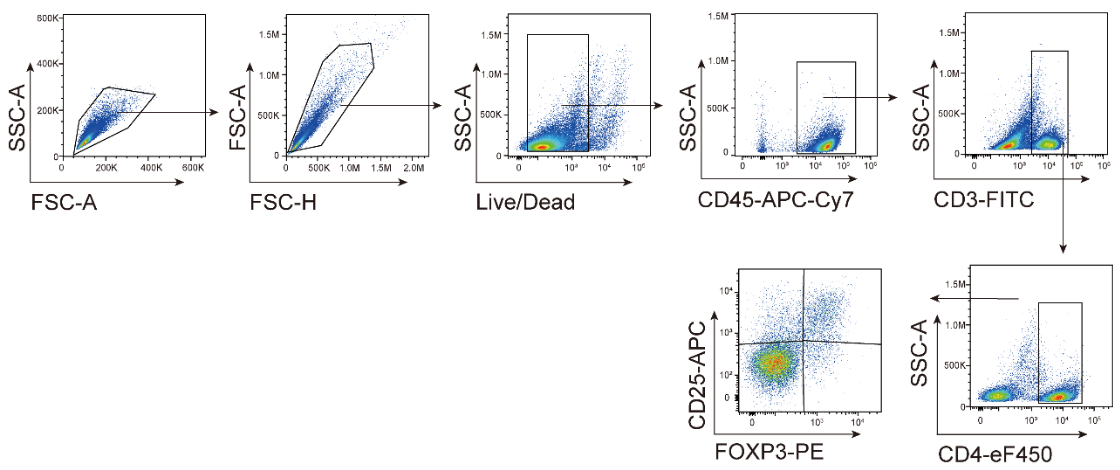

Supplementary Fig. 23. Gating strategy of Treg cells in draining lymph nodes (DLN).
