# Supplementary material for: Integration of AIEgens into covalent organic frameworks for pyroptosis and ferroptosis primed cancer immunotherapy
Source: Nat Commun. 2023 Sep 2;14:5355. doi: 10.1038/s41467-023-41121-z (PMC10475094; doi:10.1038/s41467-023-41121-z)
Supplement: Supplementary file 2 — Reporting Summary [file 41467_2023_41121_MOESM2_ESM.pdf]

nature portfolio

Corresponding author(s): Jacky W. Y. Lam, Hexiang Deng, Ben Zhong Tang, Zhi-Jun Sun

Last updated by author(s): Aug 18, 2023

Reporting Summary

Nature Portfolio wishes to improve the reproducibility of the work that we publish. This form provides structure for consistency and transparency in reporting. For further information on Nature Portfolio policies, see our Editorial Policies and the Editorial Policy Checklist.

Statistics

For all statistical analyses, confirm that the following items are present in the figure legend, table legend, main text, or Methods section.

n/a	Confirmed	
☐	☒	The exact sample size (n) for each experimental group/condition, given as a discrete number and unit of measurement
☐	☒	A statement on whether measurements were taken from distinct samples or whether the same sample was measured repeatedly
☐	☒	The statistical test(s) used AND whether they are one- or two-sided<br>*Only common tests should be described solely by name; describe more complex techniques in the Methods section.*
☐	☒	A description of all covariates tested
☐	☒	A description of any assumptions or corrections, such as tests of normality and adjustment for multiple comparisons
☐	☒	A full description of the statistical parameters including central tendency (e.g. means) or other basic estimates (e.g. regression coefficient) AND variation (e.g. standard deviation) or associated estimates of uncertainty (e.g. confidence intervals)
☐	☒	For null hypothesis testing, the test statistic (e.g. F, t, r) with confidence intervals, effect sizes, degrees of freedom and P value noted<br>*Give P values as exact values whenever suitable.*
☒	☐	For Bayesian analysis, information on the choice of priors and Markov chain Monte Carlo settings
☒	☐	For hierarchical and complex designs, identification of the appropriate level for tests and full reporting of outcomes
☒	☐	Estimates of effect sizes (e.g. Cohen's d, Pearson's r), indicating how they were calculated

Our web collection on statistics for biologists contains articles on many of the points above.

Software and code

Policy information about availability of computer code

Data collection	CytExpert software v. 2.3(Beckman), Image Studio software v. 5.2(Li-Cor Biosciences), ImageScope software v. 12.3.2 (Leica), Fotric 224s (FOTRIC), FV10-ASW v. 4.0 software (Olympus), Living Image software v.4.4 (PerkinElmer), LightField software v. 6.4 (Princeton Instruments).
Data analysis	Softwares used for analysis include GraphPad Prism v. 8.0 (GraphPad Software), FlowJo v. 10 (TreeStar), Living image software (PerkinElmer), Aperio ImageScope v. 12.3.2 (Leica), Image-Pro Plus v. 6.0 (Media Cybernetics), LightField software v. 6.4 (Princeton Instruments), Excel 2016 (Microsoft), Image Studio software v. 5.2 (Li-Cor Biosciences), CaseViewer v. 2.4 (3DHISTECH).

For manuscripts utilizing custom algorithms or software that are central to the research but not yet described in published literature, software must be made available to editors and reviewers. We strongly encourage code deposition in a community repository (e.g. GitHub). See the Nature Portfolio guidelines for submitting code & software for further information.

Data

Policy information about availability of data

All manuscripts must include a data availability statement. This statement should provide the following information, where applicable:

- Accession codes, unique identifiers, or web links for publicly available datasets
- A description of any restrictions on data availability
- For clinical datasets or third party data, please ensure that the statement adheres to our policy

The data generated in this study are available within the Article, Supplementary Information or Source Data file. Source data are provided with this paper. The full image dataset is available from the corresponding author upon request.

Human research participants

Policy information about studies involving human research participants and Sex and Gender in Research.

Reporting on sex and gender	N/A
Population characteristics	N/A
Recruitment	N/A
Ethics oversight	N/A

Note that full information on the approval of the study protocol must also be provided in the manuscript.

Field-specific reporting

Please select the one below that is the best fit for your research. If you are not sure, read the appropriate sections before making your selection.

☒ Life sciences ☐ Behavioural & social sciences ☐ Ecological, evolutionary & environmental sciences

For a reference copy of the document with all sections, see nature.com/documents/nr-reporting-summary-flat.pdf

Life sciences study design

All studies must disclose on these points even when the disclosure is negative.

Sample size	Although no sample size calculation was performed, sample sizes for the in vivo experiments are similar to those generally employed and accepted in the field (Nat Nanotechnol 14(1):89-97 (2019); Nat Biotechnol 37(11):1322-1331 (2019) and were sufficient to support our conclusions with statistical significance. Sample sizes for the in vitro experiments are also based on previous work (Nat Commun 4;12(1):6371 (2021)).
Data exclusions	No data were excluded.
Replication	All results obtained in this study were successfully replicated. Except animal experiments, we have performed the experiments shown in our manuscript more than twice. Exact numbers of biologically independent repetitions are stated in the manuscript.
Randomization	We excluded the mice that failed the model establishment. The experimental mice were grouped by random numbers table.
Blinding	Investigators were not blinded during carrying out the experiment, but blinded during the allocation, sample collection, and data analysis. In vivo experiments were performed unblinded due to the requirements and regulations of the Institutional Animal Care and Use Committee (IACUC) guidelines of Wuhan University. Bioluminescence imaging were conducted by an independent operator, who was unaware of the treatment conditions.

Reporting for specific materials, systems and methods

We require information from authors about some types of materials, experimental systems and methods used in many studies. Here, indicate whether each material, system or method listed is relevant to your study. If you are not sure if a list item applies to your research, read the appropriate section before selecting a response.

Materials & experimental systems

n/a	Involved in the study	
	x	Antibodies
	x	Eukaryotic cell lines
x		Palaeontology and archaeology
	x	Animals and other organisms
x		Clinical data
x		Dual use research of concern

Methods

n/a	Involved in the study	
x		ChIP-seq
	x	Flow cytometry
x		MRI-based neuroimaging

Antibodies

Antibodies used

The following primary antibodies were used for blocking. They are listed as antigen first, followed by supplier and clone/catalog number as applicable.
1) anti-mouse PD-1 (aPD-1), 200 μg per mouse, Bioxcell, Clone RMP1-14, Cat BE0146.

The following antibodies were used for western blotting. They are listed as antigen first, followed by supplier, catalog number as applicable.
1) anti-GAPDH, 1:5000, Proteintech, Cat 60004-1-Ig;
2) anti-GSDME, 1:1000, Abcam, Cat ab215191;
3) anti-Cleaved Caspase-3, 1:1000, Cell Signaling Technology, Cat 9664;
4) anti-GPX4, 1:1000, Abcam, Cat ab125066;
5) anti-Transferrin Receptor antibody, 1:1000, Abcam, Cat ab214039;
6) anti-xCT, 1:1000, Abmart, Cat T57046.
7) HRP-conjugated Affinipure Goat Anti-Mouse IgG(H+L), 1:5000,Proteintech, Cat SA00001-1;
8) HRP-conjugated Affinipure Goat Anti-Rabbit IgG(H+L), 1:5000,Proteintech, Cat SA00001-2;

The following antibodies were used for immunohistochemistry (IHC) and immunofluorescence. They are listed as antigen first, followed by supplier, catalog number as applicable.
1) anti-Ki67, 1:400, Abcam, Cat ab15580;
2) anti-CD8α, 1:400, Cell Signaling Technology, Cat 98941;
3) anti-4 Hydroxynonenal, 1:400, Abcam, Cat ab46545;
4) anti-granzyme B, 1:200, Cell Signaling Technology, Cat 44153;
5) anti-Cleaved Caspase-3, 1:1000, Cell Signaling Technology, Cat 9664;
6) anti-HMGB1 1:400 , Abcam, Cat ab18256;
7) Goat anti-Rabbit IgG DyLight 488 , 1:200, abbkine, Cat A23220;

The following primary antibodies were used for flow cytometry. They are listed as antigen first, followed by supplier and clone/catalog number as applicable.
1) Fixable Viability Dye, 1:1000, eBioscience, eFluor 506, Cat 65-0866-14
2) anti-mouse CD45, 1:500, eBioscience, APC-Cyanine7, Clone I3/2.3, Cat A15395;
3) anti-mouse CD3e, 1:500, eBioscience, FITC, Clone 145-2C11, Cat 11-0031-82;
4) anti-mouse CD4, 1:500, eBioscience, eFluor 450, Clone RM4-5, Cat 48-0042-82;
5) anti-mouse CD8a, 1:500, Biolegend, PerCP, Clone 5H10, Cat MCD0831;
6) anti-mouse CD11b, 1:500, Biolegend, FITC, Clone M1/70, Cat 101206;
7) anti-mouse CD11c, 1:500, eBioscience, FITC, Clone N418, Cat 11-0114-82;
8) anti-mouse CD80, 1:300, eBioscience, PE, Clone 16-10A1, Cat 12-0801-82;
9) anti-mouse CD86, 1:300, eBioscience, APC, Clone GL1, Cat 17-0862-82;
10) anti-mouse MHC-II, 1:300, eBioscience, PE-Cyanine7, Clone M5/114.15.2, Cat 25- 5321-82;
11) anti-mouse CD25, 1:200, eBioscience, APC, Clone PC61.5, Cat 17-0251-82;
12) anti-mouse Foxp3, 1:100, eBioscience, PE, Clone FJK-16s, Cat 12-5773-82;
13) anti-mouse CD44, 1:500, eBioscience, PE, Clone IM7, Cat 12-0441-82;
14) anti-mouse CD62L, 1:200, eBioscience, APC, Clone MEL-14, Cat 17-0621-82;
15) anti-mouse Ly6C, 1:200, Biolegend, APC, Clone HK1.4, Cat 128016;
16) anti-mouse Ly6G, 1:200, Biolegend, PE, Clone 1A8, Cat 127607;

Validation

No customized antibodies were used. Validation data of the antibodies purchased from commercial vendors are available on the manufactures' website and datasheets.

1) anti-mouse PD-1 (aPD-1), Bioxcell, Clone RMP1-14, Cat BE0146
https://bxcell.com/product/invivomab-anti-m-pd-1/
2) anti-GAPDH, Proteintech, Cat HRP-60004
https://www.ptgcn.com/products/GAPDH-Antibody-HRP-60004.htm
3) anti-GSDME, Abcam, Cat ab215191

https://www.abcam.cn/products/primary-antibodies/dfna5gsdme-antibody-epr19859-n-terminal-ab215191.html
4) anti-Cleaved Caspase-3, Cell Signaling Technology, Cat 9664
https://www.cellsignal.cn/products/primary-antibodies/cleaved-caspase-3-asp175-5a1e-rabbit-mab/9664
5) anti-GPX4, Abcam, Cat ab125066
https://www.abcam.cn/products/primary-antibodies/glutathione-peroxidase-4-antibody-epncir144-ab125066.html
6) anti-Transferrin Receptor antibody, Abcam, Cat ab214039
https://www.abcam.cn/products/primary-antibodies/transferrin-receptor-antibody-epr20584-ab214039.html
7) anti-xCT, Abmart, Cat T57046
http://www.ab-mart.com.cn/page.aspx?node=%2077%20&id=%202714
8) anti-Ki67, Abcam, Cat ab15580
https://www.abcam.cn/products/primary-antibodies/ki67-antibody-ab15580.html
9) anti-CD8α, Cell Signaling Technology, Cat 98941
https://www.cellsignal.com/products/primary-antibodies/cd8a-d4w2z-xp-rabbit-mab-mouse-specific/98941
10) anti-4 Hydroxynonenal, Abcam, Cat ab46545
https://www.abcam.cn/products/primary-antibodies/4-hydroxynonenal-antibody-ab46545.html
11) anti-granzyme B, Cell Signaling Technology, Cat 44153
https://www.cellsignal.com/products/primary-antibodies/granzyme-b-e5v2l-rabbit-mab-mouse-specific/44153
12) anti-mouse CD45, eBioscience, APC-Cyanine7, Clone I3/2.3, Cat A15395
https://www.thermofisher.cn/cn/zh/antibody/product/CD45-Antibody-clone-30-F11-Monoclonal/45-0451-82
13) anti-mouse CD3e, eBioscience, FITC, Clone 145-2C11, Cat 11-0031-82
https://www.thermofisher.cn/cn/zh/antibody/product/CD3e-Antibody-clone-145-2C11-Monoclonal/11-0031-82
14) anti-mouse CD4, eBioscience, eFluor 450, Clone RM4-5, Cat 48-0042-82
https://www.thermofisher.cn/cn/zh/antibody/product/CD4-Antibody-clone-RM4-5-Monoclonal/48-0042-82
15) anti-mouse CD8a, eBioscience, PerCP, Clone 5H10, Cat MCD0831
https://www.thermofisher.cn/cn/zh/antibody/product/CD8-alpha-Antibody-clone-5H10-Monoclonal/MCD0831
16) anti-mouse CD11b, Biolegend, FITC, Clone M1/70, Cat 101206
17) anti-mouse CD11c, eBioscience, FITC, Clone N418, Cat 11-0114-82
https://www.thermofisher.cn/cn/zh/antibody/product/CD11c-Antibody-clone-N418-Monoclonal/11-0114-82
https://www.biolegend.com/en-us/products/fitc-anti-mouse-human-cd11b-antibody-347
18) anti-mouse CD80, eBioscience, PE, Clone 16-10A1, Cat 12-0801-82
https://www.thermofisher.cn/cn/zh/antibody/product/CD80-B7-1-Antibody-clone-16-10A1-Monoclonal/12-0801-82
19) anti-mouse CD86, eBioscience, APC, Clone GL1, Cat 17-0862-82
https://www.thermofisher.cn/cn/zh/antibody/product/CD86-B7-2-Antibody-clone-GL1-Monoclonal/17-0862-82
20) anti-mouse MHC-II, eBioscience, PE-Cyanine7, Clone M5/114.15.2, Cat 25-5321-82
https://www.thermofisher.cn/cn/zh/antibody/product/MHC-Class-II-I-A-I-E-Antibody-clone-M5-114-15-2-Monoclonal/25-5321-82
21) anti-mouse CD25, eBioscience, APC, Clone PC61.5, Cat 17-0251-82
https://www.thermofisher.cn/cn/zh/antibody/product/CD25-Antibody-clone-PC61-5-Monoclonal/17-0251-82
22) anti-mouse Foxp3, eBioscience, PE, Clone FJK-16s, Cat 12-5773-82
https://www.thermofisher.cn/cn/zh/antibody/product/FOXP3-Antibody-clone-FJK-16s-Monoclonal/12-5773-82
23) anti-mouse CD44, eBioscience, PE, Clone IM7, Cat 12-0441-82
https://www.thermofisher.cn/cn/zh/antibody/product/CD44-Antibody-clone-IM7-Monoclonal/12-0441-82
24) anti-mouse CD62L, eBioscience, APC, Clone MEL-14, Cat 17-0621-82
https://www.thermofisher.cn/cn/zh/antibody/product/CD62L-L-Selectin-Antibody-clone-MEL-14-Monoclonal/17-0621-82
25) anti-mouse Ly6C, Biolegend, APC, Clone HK1.4, Cat 128016
https://www.biolegend.com/en-us/products/apc-anti-mouse-ly-6c-antibody-6047
26) anti-mouse Ly6G, Biolegend, PE, Clone 1A8, Cat 12760730) rabbit IgG isotype control, Cell Signaling Technology, Alexa Fluor 488, Cat 2975
https://www.biolegend.com/en-us/products/pe-anti-mouse-ly-6g-antibody-4777
27) HRP-conjugated Affinipure Goat Anti-Mouse IgG(H+L), Proteintech, Cat SA00001-1https://www.ptgcn.com/products/HRP-conjugated-Affinipure-Goat-Anti-Mouse-IgG-H-L-secondary-antibody.htm
28) HRP-conjugated Affinipure Goat Anti-Rabbit IgG(H+L), Proteintech, Cat SA00001-2https://www.ptgcn.com/products/HRP-conjugated-Affinipure-Goat-Anti-Rabbit-IgG-H-L-secondary-antibody.htm
29) Goat anti-Rabbit IgG DyLight 488, Abbkine, Cat A23220 https://www.abbkine.cn/product/a23220/
30) Fixable Viability Dye, 1:1000, eBioscience, Cat 65-0866-14
https://www.thermofisher.cn/order/catalog/product/65-0866-14?SID=srch-srp-65-0866-14

Eukaryotic cell lines

Policy information about cell lines and Sex and Gender in Research

Cell line source(s)	The murine breast cancer cell line 4T1 (Cat CRL-2539) was purchased from the American Type Culture Collection (ATCC). 4T1-Luc cell line was obtained from Prof. Xian-Zheng Zhang at Wuhan University. The original commercial source of the 4T1-Luc cell line was ATCC.
Authentication	Each cell line used was morphologically confirmed according to the information provided by culture collections.
Mycoplasma contamination	All the cell lines presented in this study were tested for mycoplasma contamination and they were free of mycoplasma contamination.

Commonly misidentified lines (See ICLAC register)	No commonly misidentified cell lines were used.

Animals and other research organisms

Policy information about studies involving animals; ARRIVE guidelines recommended for reporting animal research, and Sex and Gender in Research

Laboratory animals	The female BALB/c mice (6–8 weeks) were provided by the Experimental Animal Central of Wuhan University.
Wild animals	The study did not involve wild animals.
Reporting on sex	Only female mice were used in this study.
Field-collected samples	The study did not involve samples collected from field.
Ethics oversight	Ethical approval of this study was obtained from the Institutional Animal Care and Use Committee (IACUC) guidelines of Wuhan University (approval number: WP20220030). All animal experimental procedures were performed in accordance with the Regulations for the Administration of Affairs Concerning Experimental Animals approved by the State Council of the People's Republic of China.

Note that full information on the approval of the study protocol must also be provided in the manuscript.

Flow Cytometry

Plots

Confirm that:

- [x] The axis labels state the marker and fluorochrome used (e.g. CD4-FITC).
- [x] The axis scales are clearly visible. Include numbers along axes only for bottom left plot of group (a 'group' is an analysis of identical markers).
- [x] All plots are contour plots with outliers or pseudocolor plots.
- [x] A numerical value for number of cells or percentage (with statistics) is provided.

Methodology

Sample preparation	For lymphocyte analysis, splenocytes and tumor-draining lymph nodes were collected from mice and made into cell suspensions by gentleMACS™ dissociator and digestive enzyme (Miltenyi Biotec) according to the manufacturer's instructions. Then, the samples were passed through 200-mesh nylon mesh filters to obtain single-cell suspensions. Live or dead cells were separated by Fixable Viability Dye (eBioscience, Dye eFluor 506). For all samples, cells were first stained with antibodies against surface antigens. In some experiments, cells were subsequently fixed, permeablized and stained for intracellular antigens.
Instrument	CytoFLEX flow cytometer (Beckman), MoFlo XDP cell sorter (Beckman)
Software	CytExpert v. 2.3 software (Beckman), FlowJo v. 10 (TreeStar)
Cell population abundance	The purity of the post-sorted cells was more than 95% as verified by flow cytometry.
Gating strategy	The gating strategy was shown in Supplementary Fig. 15-18. Generally, single cell gates based on SSC-H and SSC-A, and FSC-H and FSC-A were used to exclude non-singlets. A live/dead cell gate based on fixable viability dye was used to exclude dead cells.

- [x] Tick this box to confirm that a figure exemplifying the gating strategy is provided in the Supplementary Information.
